# What do we mean by structural determinants of health, why is it important, and are public health communities ready to engage?

**DOI:** 10.17269/s41997-025-01122-5

**Published:** 2025-11-13

**Authors:** Lindsay McLaren, Elizabeth McGibbon

**Affiliations:** 1 University of Calgary, Calgary, AB Canada; 2 St. Francis Xavier University, Antigonish, NS Canada

The phrase *structural determinants of health* (structural DoH) is gaining prominence in Canadian public health discourse (e.g., Clark et al., 2022; Heller et al., 2024; CIHR, 2024). According to an important new book (McGibbon, 2024), structural DoH are the systemic or root causes of health inequities and injustices across all life on Earth and the health of the Earth itself.

Public health, the art and science of preventing sickness and promoting health through organized efforts of society, is concerned with keeping people healthy in the first place (CPHA, n.d.). Structural DoH are therefore firmly within public health’s wheelhouse, or they should be. As our field drifts further downstream (McLaren et al., 2024; Yong, 2021) and becomes increasingly indistinguishable from downstream medical care in some contexts (Haines-Saah and McLaren, 2023), it is imperative that public health communities engage meaningfully with this evolving dialogue. How might we ensure that we do so?

It is helpful to first consider the reasons for the newer language. As Heller et al. (2024) and others discuss, the previously popular concept, *social DoH,* has been used reductively, for example, to refer to decontextualized living and working conditions or to static notions of gender or race, such that social DoH are disconnected from structural change. In other words, the *social DoH* have been depoliticized. This, it is argued, signals a need for a focus on structural DoH, with clear definitions.

While clear definitions are appealing, we urge critical reflection. Structural DoH, McGibbon (2024) argues, demand engagement with systemic power relations and their roles in creating and perpetuating inequities in the factors shaping health—including who controls dominant narratives and who benefits from economic and social inequality. Structural DoH are thus inherently *emergent*. Fixating on definitions, which tends to promote static and reductive thinking, risks obscuring the complex dynamics shaping health and its distribution.

Moreover, it bears reminding that social DoH *could*, and indeed should, be politicized. The milestone work of the WHO Commission on Social Determinants of Health, for example, applies a structural lens when noting that health inequities reflect “a toxic combination of poor social policies and programmes, unfair economic arrangements, and bad politics”, and that closing the health equity gap will require “tackling the inequitable distribution of power, money, and resources” (CSDH 2008). Raphael (2009) has long argued that the study of the social DoH concerns two key problems: the conditions in which people live, work, and play (the social *factors*); *and* the social, economic, and political forces that shape the quality and (inequitable) distribution of those conditions (societal *forces*).

Alongside and often within *structural DoH,* other categories (e.g., commercial/corporate determinants, political-economic determinants) have emerged, raising questions about their added analytic and activist value. Indeed, some scholars have argued for the continued importance of *social DoH* as an overarching label, *so long as those using the term are thinking relationally and within critical social perspectives*. Muntaner and Benach (2023) make this point with reference to the Weberian tradition in sociology, which distinguishes three types of *social* relations, all of which are highly pertinent to structural DoH equity: economic (relations of production, appropriation, and distribution), political (relations of power), and cultural (relations of equivalence, such as speaking the same language).

The point is that the *phrase itself* is not the problem, it is how the phrase has been interpreted and used, which itself reflects the very power dynamics that must be foregrounded if we are serious about supporting the public’s health. Changing the language will not help us think and act structurally unless we engage with the reasons why we have depoliticized other radical concepts. Furthermore, lack of action is not due to definitional problems—it is persistent neoliberal resistance to equity-based discourse in public policy (Raphael & Bryant, 2023).

## Structural determinants of health: The imperative of critical perspectives

Are Canadian public health communities ready to engage with structural DoH, including its inherent criticality? We consider this question from our perspectives as critical scholars with expertise in structural – especially political-economic—determinants of health equity. We also both served as members of the peer review committee (one of us as Scientific Officer) for the recent CIHR Institute of Population and Public Health funding opportunity on *Moving upstream: structural determinants of health*.

Our experiences have provided some important insights into our community’s readiness to engage with structural DoH. First, it is fair to say that there is *not* presently a shared understanding of what is meant by structural DoH, including its value commitments. Members of our communities bring, for example, different perspectives around how or where equity fits in this space, and whether or not structural DoH infer attention to the beneficiaries of capital accumulation and exploitation. While variations in perspectives can be a strength, it can also be a problem if it serves to silence or sanitize critical voices, which must be at the heart of a structural determinants orientation.

Second, there seems to be, so far, a strong emphasis on how structural determinants operate in health care contexts. Health care is important, and it is certainly possible to think and act structurally about health care and peoples’ interactions with it. However, this prominence raises the question of whether we are forgetting an impactful crux of the social DoH: that health care, with its overwhelmingly downstream orientation, has little to do with who gets sick in the first place. If we envision structural determinants language as a way to advance upstream thinking in our field, then this trend is a step backwards.

Whether public health communities are ready to engage constructively with structural DoH depends, we argue, on the willingness to think and act *critically*. As one helpful heuristic for doing so, Green and Labonté (2008; see also McLaren et al., 2025) discuss the importance of a critical voice *for* public health, and a critique *of* public health.

A critical voice *for* public health means a stance for uncovering the health damaging effects of particular social structures, in their political and historical contexts; and challenging those structures. Speaking to the essential normative elements, this includes the obligation to call out when certain values, such as the interests of business, trump others such as health and equity (Green & Labonté, 2008). Significantly, ‘calling out’ requires overcoming a resistance to engaging with “Big P” politics (e.g., Right / alt-Right or Left, socialist leanings) and naming perpetrators, including those in political leadership (McGibbon, 2024).

A critique *of* public health, on the other hand, involves asking difficult questions about our field and constantly questioning our own assumptions. This requires reflection, from a place of humility, about the ways that we benefit from and are complicit in the status quo, both as individuals, who may be well paid professionals with secure employment, and as a field (Green & Labonté, 2008).

A recent analysis yields important insights from the point of view of a critique *of* public health. Inspired by the now well-established connections among political economy, population well-being, and health equity (i.e., structural DoH), McLaren and Mykhalovskiy (2024) analyzed the Canadian public health community’s historical engagement with economic policy, as gleaned through the pages of this journal. We observed a strong and seemingly subconscious allegiance to dominant economic paradigms (capitalism), despite their incompatibility with the public’s health including health equity. And, although CJPH authors have long been preoccupied with socio-economic inequalities in health, we observed that those inequalities are consistently decoupled from their roots in political economic systems. These strong patterns are at odds with, and will significantly challenge, our field’s efforts to engage structurally.

These observations are not limited to the past. We both have experience trying to incorporate discussion of capitalism into mainstream health contexts, only to be (rather aggressively) met with responses along the lines of, “that’s interesting, but *in the real world* …”, signalling that this structural paradigm, which dramatically shapes relations of power, somehow lies outside of the core activities of public health. More broadly, we both routinely experience being disrespected or actively dismissed by those working in mainstream spaces: we are not considered to be a ‘real’ part of the field, either because we don’t work in practice and therefore don’t understand ‘the real world’, or are considered too low in the professional clinical hierarchy to have any credibility. If our field is to have any coherence around our shared goal of supporting the public’s health, respectful and constructive relationships between mainstream and critical perspectives are essential (Mykhalovskiy et al., 2019).

Overall, to engage meaningfully and constructively with the evolving emphasis on structural DoH, and to resist the depoliticization that has historically occurred with other radical terms, we need to do more than change our language and seek ever-elusive “correct” definitions – we need to figure out how to engage critically as a field. We hope that this editorial will prompt and support ongoing conversations in this space.

*L’expression déterminants structurels de la santé* (DS structurels) figure de plus en plus dans le discours de la santé publique canadienne (p. ex. Clark et al., 2022; Heller et al., 2024; CIHR, 2024). Selon un nouveau livre important (McGibbon, 2024), les DS structurels sont les causes systémiques ou fondamentales des iniquités et des injustices qui troublent la santé de toute vie sur Terre et de la Terre elle -même.

La santé publique, la science et l’art de prévenir les maladies et de promouvoir la santé au moyen d’une action sociétale organisée, se préoccupe en premier lieu de garder les gens en bonne santé (CPHA, n.d.). Les DS structurels sont donc tout à fait dans ses cordes – ou du moins, ils devraient l’être. À l’heure où la santé publique dérive en aval (Yong, 2021; McLaren et al., 2024) et où il devient de plus en plus difficile de la distinguer des soins médicaux dans certains contextes (Haines-Saah & McLaren, 2023), il faut impérativement que les communautés qui la composent participent véritablement à ce dialogue en évolution. Comment nous en assurer?

Il est utile de commencer par nous pencher sur les raisons de cette nouvelle expression. Comme le mentionnent Heller et collègues (2024) et d’autres, le concept autrefois populaire des déterminants sociaux de la santé (*DS sociaux*) a été utilisé de façon réductrice, par exemple pour désigner les conditions de vie et de travail hors de leur contexte ou des notions statiques du genre ou de la race, détachant ainsi les DS sociaux des changements structurels. Autrement dit, les *DS sociaux* ont été dépolitisés. Cela, fait-on valoir, signale qu’il faut mettre l’accent sur les DS structurels, avec des définitions claires.

Malgré l’attrait des définitions claires, nous appelons à une réflexion critique. Les DS structurels, soutient McGibbon (2024), exigent que l’on s’intéresse aux relations de pouvoir systémiques et à leur rôle dans la création et la pérennisation d’iniquités dans les facteurs de la santé, y compris aux acteurs qui contrôlent le discours dominant et à ceux qui bénéficient des inégalités économiques et sociales. Les DS structurels sont donc implicitement *émergents*. La fixation sur les définitions, qui tend à promouvoir une pensée statique et réductrice, risque d’obscurcir la dynamique complexe de la santé et de sa répartition.

Rappelons du reste que les DS sociaux *pourraient*, et à vrai dire devraient, être politisés. Dans une publication phare, la Commission des déterminants sociaux de la santé de l’OMS applique par exemple un prisme structurel en disant que les iniquités en santé résultent «des effets conjugués de politiques et de programmes sociaux insuffisants, de modalités économiques injustes et de stratégies politiques mal pensées», et que pour les combler, il faut «lutter contre les inégalités dans la répartition du pouvoir, de l’argent et des ressources» (CSDH, 2008). Raphael (2009) soutient depuis longtemps que l’étude des DS sociaux porte sur deux grands problèmes: les conditions dans lesquelles les gens vivent, travaillent et jouent (les facteurs sociaux); *et* les forces sociales, économiques et politiques qui structurent la qualité et la répartition (inéquitable) de ces conditions (les *forces* sociétales).

En plus et souvent au centre des *DS structurels*, d’autres catégories (les déterminants commerciaux/corporatifs, les déterminants politico-économiques) commencent à émerger, ce qui soulève des questions sur leur valeur ajoutée pour l’analyse et le militantisme. À vrai dire, certains théoriciens défendent l’importance continue des *DS sociaux* comme étiquette générale, *à condition que les utilisateurs du terme aient une pensée relationnelle qui s’inscrit dans une perspective sociale critique*. Muntaner & Benach (2023) font valoir ce point au sujet de la tradition wébérienne en sociologie, qui distingue trois types de relations *sociales*, toutes très pertinentes pour l’équité des DS structurels: les relations économiques (de production, d’appropriation, de répartition), les relations politiques (de pouvoir) et les relations culturelles (d’équivalence, comme le fait de parler la même langue). 

Le fait est que ce n’est pas *l’expression même* qui pose un problème, mais bien son interprétation et son utilisation, ce qui reflète en soi la dynamique de pouvoir qu’il faut absolument mettre au premier plan si nous voulons vraiment favoriser la santé publique. Un simple changement de vocabulaire ne nous aidera pas à avoir une pensée et une action structurelles; il faudra pour cela nous intéresser aux raisons pour lesquelles nous avons dépolitisé d’autres concepts radicaux. D’autre part, le manque d’action n’est pas dû à un problème de définition, mais à la résistance néolibérale persistante au discours d’équité dans les politiques publiques (Raphael & Bryant, 2023).

## Les déterminants structurels de la santé: la nécessité impérieuse d’une perspective critique

La communauté de la santé publique canadienne est-elle prête à s’intéresser aux DS structurels, y compris à leur criticité implicite? Nous envisageons la question de notre point de vue de spécialistes de la théorie critique, en particulier des déterminants structurels – surtout politico-économiques – de l’équité en santé. Nous avons aussi toutes les deux siégé au comité d’évaluation par les pairs (l’une en tant qu’agente scientifique) d*’Agir en amont: subventions Catalyseur sur les déterminants structurels de la santé*, la récente possibilité de financement de l’Institut de la santé publique et des populations des IRSC.

Notre expérience apporte un éclairage utile sur la volonté de la communauté de la santé publique de s’intéresser aux DS structurels. Premièrement, il est juste de dire qu’il n’y a pas actuellement de conception commune de ce que l’on veut dire par les DS structurels, ce qui inclut leurs engagements moraux. Les membres de nos communautés, par exemple, voient différemment comment et où l’équité s’inscrit dans cet espace et si les DS structurels supposent ou non que l’on s’intéresse aux bénéficiaires de l’accumulation du capital et de l’exploitation. Cette diversité de points de vue peut être une force, mais elle peut aussi être un problème si elle sert à faire taire ou à aseptiser les voix critiques, qui doivent être au coeur d’une orientation sur les déterminants structurels.

Deuxièmement, on semble insister fortement jusqu’ici sur le fonctionnement des déterminants structurels dans les milieux des soins de santé. Les soins de santé sont importants, et il est certainement possible de penser et d’agir de façon structurelle à leur sujet et au sujet des interactions qu’ont les gens avec ce type de soins. Leur prééminence soulève toutefois la question de savoir si nous oublions l’une des raisons d’être des DS sociaux: le fait que les soins de santé, très majoritairement orientés vers l’aval, se soucient peu de savoir qui tombe malade en premier lieu. Si nous envisageons le vocabulaire des déterminants structurels comme un moyen de faire progresser la pensée en amont dans notre domaine, une telle tendance représente un recul.

Quant à savoir si les communautés de la santé publique sont prêtes à aborder les DS structurels de façon constructive, cela dépend, selon nous, de leur volonté d’avoir une pensée et une action *critiques*. Comme méthode heuristique utile pour y parvenir, (Green et Labonté 2008; voir aussi McLaren, Green, & Labonté, 2025) discutent de l’importance d’une voix critique *pour* la santé publique et d’une critique *de* la santé publique.

Une voix critique *pour* la santé publique, c’est une position qui vise à mettre en lumière les effets néfastes sur la santé de structures sociales particulières, dans leur contexte politique et historique; et à remettre ces structures en question. Les éléments normatifs essentiels de la santé publique incluent l’obligation de signaler quand certaines valeurs, comme les intérêts des entreprises, l’emportent sur d’autres, comme la santé et l’équité (Green & Labonté, 2008). Or, de tels signalements exigent que l’on surmonte sa résistance à faire de la «politique» (la droite, l’extrême-droite, la gauche, les penchants socialistes) et que l’on nomme les agresseurs, même si ce sont des dirigeants politiques (McGibbon, 2024).

Une critique *de* la santé publique, en revanche, consiste à poser des questions difficiles sur notre domaine et à remettre sans cesse nos suppositions en question. Cela demande une humble réflexion sur les façons dont nous bénéficions du statu quo et dont nous en sommes complices, tant individuellement, comme professionnels bien rémunérés qui jouissons d’un emploi sûr, que collectivement (Green & Labonté, 2008).

Une analyse récente apporte un éclairage important du point de vue d’une critique *de* la santé publique. Inspirés par les liens aujourd’hui bien établis entre l’économie politique, le bien-être des populations et l’équité en santé (c.-à-d. les DS structurels), McLaren et Mykhalovskiy (2024) ont analysé l’intérêt historique de la communauté de la santé publique canadienne envers la politique économique d’après les informations glanées dans les pages de la *Revue canadienne de santé publique*. Nous avons nous-mêmes observé une allégeance solide et apparemment inconsciente envers le paradigme économique dominant (le capitalisme), en dépit de son incompatibilité avec la santé du public, dont l’équité en santé. Et bien que les auteurs et autrices de la RCSP se préoccupent depuis longtemps des inégalités socioéconomiques en santé, nous avons observé que ces inégalités sont systématiquement dissociées de leurs racines dans les systèmes politico-économiques. Ces tendances marquées sont en décalage avec les efforts d’engagement structurel déployés dans notre domaine et les mettent sérieusement en cause.

Ces observations ne se limitent pas au passé. Nous avons toutes les deux essayé d’intégrer une discussion du capitalisme dans les contextes de santé dominants pour nous heurter à des réponses (plutôt agressives) comme «c’est intéressant, mais *dans le monde réel*…», qui laissent entendre que ce paradigme structurel, qui modèle considérablement les relations de pouvoir, se trouve curieusement en dehors des activités de base de la santé publique. Plus généralement, nous avons toutes les deux été régulièrement méprisées ou activement écartées par des personnes qui travaillent les espaces dominants: nous ne sommes pas considérées comme faisant «vraiment» partie du domaine, soit parce que nous ne sommes pas praticiennes, et que de ce fait nous ne comprenons pas «le monde réel», soit parce qu’on nous voit comme étant trop bas dans la hiérarchie clinique professionnelle pour avoir quelque crédibilité que ce soit. Pour que notre domaine affiche une certaine cohérence dans son objectif commun de favoriser la santé publique, il est indispensable qu’il y ait des liens respectueux et constructifs entre le point de vue dominant et les opinions critiques (Mykhalovskiy et al. 2019).

Globalement, pour aborder utilement et de façon constructive l’adoption croissante de la notion des DS structurels et pour résister à la dépolitisation dont d’autres termes radicaux ont fait l’objet par le passé, il faudra faire plus que de changer notre vocabulaire et de pourchasser les définitions «correctes» (qui nous échapperont toujours): il faudra trouver comment amener tout notre domaine à engager une réflexion critique. Nous espérons que cet éditorial incitera et appuiera des discussions continues dans cet espace.

## Data Availability

Not applicable.

## References

[CR1] Canadian Institutes of Health Research (CIHR). (2024). Institute of Population and Public Health. Catalyst grant: Moving upstream: Structural determinants of health (archived), https://cihr-irsc.gc.ca/e/53883.html

[CR2] Canadian Public Health Association (CPHA). (n.d.) What is public health? Available here (accessed July 3, 2025).

[CR3] Clark, E. C., Cranston, E., Polin, T., Ndumbe-Eyoh, S., MacDonald, D., Betker, C., & Dobbins, M. (2022). Structural interventions that affect racial inequities and their impact on population health outcomes: A systematic review. *BMC Public Health,**22*, 2162. 36424559 10.1186/s12889-022-14603-w PMC9685079

[CR4] Commission on Social Determinants of Health (CSDH). (2008). *Closing the gap in a generation: Health equity through action on the social determinants of health*. World Health Organization. 19385432

[CR5] Green, J., & Labonté, R. (2008). *Critical perspectives in public health*. Routledge.

[CR6] Haines-Saah, R., & McLaren, L. (2023). A cautionary tale: University institutes of public health must “walk the talk.” *Canadian Journal of Public Health,**114*(5), 710–713. 37644254 10.17269/s41997-023-00815-z PMC10484852

[CR7] Heller, J. C., Givens, M. L., Johnson, S. P., & Kindig, D. A. (2024). Keeping it political and powerful: Defining the structural determinants of health. *The Milbank Quartly,**102*(2), 0227. 10.1111/1468-0009.12695 PMC11176401 38363858

[CR8] McGibbon, E. (2024). *The politics of social, ecological, and structural determinants of health in Canada*. Canadian Scholars.

[CR9] McLaren, L., & Mykhalovskiy, E. (2024). Economic policy and public health: Insights from the history of the Canadian Journal of Public Health. *Canadian Journal of Public Health,**115*(5), 705–719. 39436542 10.17269/s41997-024-00940-3 PMC11535091

[CR10] McLaren, L., Juzwishin, D. W. M., & Velez Mendoza, R. (2024). *A history of public health in Alberta, 1919–2019*. University of Calgary Press.

[CR11] McLaren, L., Green, J., & Labonté, R. (2025). New directions in critical public health: Health in turbulent times. *Routledge*. 10.4324/9781003654650

[CR12] Muntaner, C., & Benach, J. (2023). Why social (political, economic, cultural, ecological) determinants of health? Part 1: Background of a contested construct. *International Journal of Social Determinants of Health and Health Services,**53*(2), 117–121. 36721356 10.1177/27551938231152996

[CR13] Mykhalovskiy, E., Frohlich, K. L., Poland, B., Di Ruggiero, E., Rock, M. J., & Comer, L. (2019). Critical social science *with* public health: Agonism, critique and engagement. *Critical Public Health 2019,**29*(5), 522–533.

[CR14] Raphael, D. (Ed.). (2009). *Social determinants of health: Canadian perspectives* (2nd ed.). Canadian Scholars’ Press Inc.

[CR15] Raphael, D., & Bryant, T. (2023). Socialism as the way forward: Updating a discourse analysis of the social determinants of health. *Critical Public Health,**33*(4), 387–394.

[CR16] Yong, E. (2021). *How public health took part in its own downfall*. The Atlantic.

